# OLDER MEDICARE BENEFICIARIES WITH KIDNEY FAILURE FROM PUERTO RICO MIGRATED AFTER HURRICANE MARIA

**DOI:** 10.1093/geroni/igad104.1761

**Published:** 2023-12-21

**Authors:** Maricruz Rivera-Hernandez

**Affiliations:** Brown University School of Public Health, Providence, Rhode Island, United States

## Abstract

The vast majority of the literature regarding migration often focuses on younger and healthy migrants. However, recent studies have shown that older adults with complex chronic conditions are migrating to receive services and supports from family members, especially after natural disasters. After Hurricane Maria landed in Puerto Rico in 2017, health care facilities struggled to provide care. Patients with complex chronic conditions faced significant challenges to receive care. The objective of this study is to characterize migration rates among older Medicare beneficiaries with kidney failure after Hurricane Maria. We conducted a cross-sectional analysis of older Medicare beneficiaries (65 and over) with kidney failure from Puerto Rico from 2010 to 2020. We used data from the Master Beneficiary Summary File and the Chronic Conditions Data Warehouse (CCW) Chronic Conditions Segment. Beneficiaries with kidney failure were identified using the End-Stage Renal Disease Indicator from the CCW. Outmigration was defined as changing the state of residence the following year. There were 9450 which contributed 28445 person-years of information. About 537 (1.9%) older adults with kidney failure migrated to the US mainland during the study period. Migration rates before Maria were 1.5 (95% CI, 1.3 – 1.7), which increased by 3.6 percentage points (95% CI, 2.8 – 4.4). However, migration rates dropped to pre-Maria levels after 2017. Older migrants were more likely to be females, fee-for-service beneficiaries and younger compared to non-migrants. These findings suggest that older adults with kidney failure migrated to the US mainland to access care immediately after Hurricane Maria.

